# Effect of platelet‐rich plasma and platelet‐rich fibrin on healing of burn wound with dual‐species biofilm

**DOI:** 10.1002/kjm2.12940

**Published:** 2025-01-19

**Authors:** Wen‐Dan Li, Fang Lin, Yu Sun, Zi‐Jing Zhu, Mei‐Liang Luo, Yi‐Qi Zeng, Zhen Lin, Mou Zhou

**Affiliations:** ^1^ Department of Blood Transfusion General Hospital of Southern Theatre Command of PLA Guangzhou City, Guangdong Province China

**Keywords:** bacteria, burn wound, dual‐species biofilm, platelet‐rich fibrin, platelet‐rich plasma

## Abstract

This study evaluated the impact of platelet‐rich plasma (PRP) and platelet‐rich fibrin (PRF) on burn wound with dual‐species biofilm. *Pseudomonas aeruginosa* (*P. aeruginosa*) and *Staphylococcus aureus* (*S. aureus*) were applied to infect the burn wound in rats to establish a dual‐species biofilm model. After infection, the wound was treated with ionized silver (AG), PRF, and PRP. Silver scanning electron microscopy (SEM) was used to assess adhesion after infection. PRF and PRP reduced wound size from day 8 after burn injuries, while AG significantly promoted burn wound healing at day 12. New collagen was formed in the shortest time in PRF and PRP groups compared to AG and control groups. PRF and PRP greatly lowered the bacterial numbers in wounds with *S. aureus* and *P. aeruginosa* biofilm, whereas AG showed weak bacteriostatic effects. AG, PRF, and PRP treatments significantly reduced inflammatory mediators and induced VEGFA. However, AG treatment increased TNF‐α. PRF and PRP accelerate wound healing in the presence of dual‐species biofilm infection and show strong antibacterial activity against *S. aureus* and *P. aeruginosa*, indicating that PRF and PRP could be potential therapies for burn wounds with dual‐species biofilm infection.

## 1 INTRODUCTION

Bacterial infection contributes to most death in burn patients, accounting for 75% of all burn deaths. *Pseudomonas aeruginosa* (*P. aeruginosa*), *Acinetobacter baumannii* (*A. baumannii*), and *Staphylococcus aureus* (*S. aureus*), are notorious for colonizing burn wounds. Biofilms are organized communities of mono or multispecies bacteria, usually manifesting as bacterial aggregates.^1^ Biofilm‐grown bacteria are thought to develop antimicrobial resistance compared to planktonic bacteria.^2^ The presence of biofilm infection is the cause of 65% of chronic wound healing impairments.^3^ As a result, bacteria in biofilms are difficult to remove from wounds, and bacterial biofilms are a major challenge to wound healing. Furthermore, dual‐species biofilms show higher biomass/metabolic activity/CFU.^4^ Therefore, new approaches for the treatment of biofilm infections are needed, especially for dual‐ or multiply‐biofilm infections.

Silver is being used as a new treatment for biofilm infections, with less impact on antibiotic‐resistant bacteria than antibiotics.^5^ Kostenko et al.^6^ reported the antimicrobial efficacy of silver on mono‐species biofilm. However, data on the impact of silver on dual‐species biofilms in burn wound in vivo are limited.

In recent years, platelet‐rich plasma (PRP) can promote wound healing process.^7^ Platelets in PRP can secrete several factors, including vascular endothelial growth factor (VEGF), basic fibroblast growth factor (bFGF), which play a crucial role in the healing process.^7^, ^8^ PRP has been reported to inhibit the growth of wound pathogens.^9^

Platelet‐rich fibrin (PRF) contains microfibrillar proteins containing platelets and leukocytes and is more advanced than conventionally prepared PRP.^10^ PRF is widely used for dentistry or oral maxillofacial surgery.^11^ Jasmine et al. have reported that PRF possesses antimicrobial and antibiofilm effects on staphylococcus bacteria in vitro.^10^ However, its antimicrobial effects on dual‐biofilm in burn wound have not been investigated.

*S. aureus* and *P. aeruginosa* are the main pathogens that grow together in biofilms.^1^ Therefore, we established a dual‐species biofilm model community of *S. aureus* and *P. aeruginosa* after burns in rats to assess the effect of PRF/PRP on wound healing. We found that PRP and PRF had antimicrobial effects on dual‐species biofilms of burn wounds and accelerated wound healing in rats. Even ionic silver (AG) promoted wound healing, but its antimicrobial effect on dual‐species biofilms was poor.

## 2 METHODS

### 2.1 Animals

A total of 24 three‐month‐old male Sprague–Dawley rats were used in this study. Animals were housed in individual cages at a constant temperature (25°C) with a 12‐h light/12‐h dark cycle and provided with food and water.

### 2.2 Creation of burn wounds

Full‐thickness burns were performed on the backs of 40 male Sprague–Dawley rats as previously described.^12^ Briefly, the rats were weighed and anesthesia was provided by intraperitoneal injection of formulated 3.6% chloral hydrate at a dosage of 10 mL/kg. The hair on the back of the rats was moistened with soft soap solution and then carefully shaved off. A circular brass disk 2 cm in diameter was heated to 88–90°C for 20 s without applying any external pressure to achieve burn injury. The middle of the back of the rat's shaved area was exposed to this burn mold. Each rat had burns on both sides.

### 2.3 PRF and PRP preparation

After anesthetizing the rats, the abdominal cavity was opened along the mid‐abdominal line, and 10 mL of blood was taken from the main abdominal vein and quickly put into a centrifuge tube. The blood was centrifuged at 3000 rpm for 10 min at room temperature using a sterile centrifuge tube without any anticoagulant or procoagulant. After centrifugation, the blood was divided into three layers, and the yellowish gel‐like substance in the middle layer was rat PRF, which was placed in a sterile test tube with the erythrocyte component subtracted from the tail end, and then stored at −80°C.

Venous blood (7 mL) was withdrawn using a vacuum blood collection tube and centrifuged at 1500 rev/min for 20 min. The upper layer of plasma and 1 mm of erythrocytes at the stratification interface were aspirated with a pasteurized tube, and the remaining blood was centrifuged at 2000 rev/min for 10 min. Platelets were deposited in the bottom white layer, and after the upper plasma was aspirated, 0.5 mL of plasma and blood cell fraction were left to stand for 30 min, after which the tube was gently shaken until the erythrocytes and platelets were resuspended in the plasma. The extracted PRP was stored at 4°C.

### 2.4 Formation of *P. aeruginosa* and *S. aureus* biofilm

The *P. aeruginosa* standard strain was obtained from ATCC9027 and the *S. aureus* standard strain was obtained from ATCC25923. Single‐colony *S. aureus* and *P. aeruginosa* were grown in trypticase soy broth (TSB) medium. Briefly, a 1:1 mixture of 10 mL of culture (*P. aeruginosa* and *S. aureus* biofilm bacterial loads were both 1 × 10^7^ CFU/mL) was inoculated into tubes. *S. aureus* and *P. aeruginosa* were applied evenly to the burn wounds daily for 4 days. The rats were randomly assigned into (1) Control (without treatment), (2) AG group, (3) PRF group, and (4) PRP, each group with 10 rats. The dressing was applied as a smear to treat the burn wound sites of rats individually. The dressing should be changed every 4 days and 0.3 mL should be used at a time.

### 2.5 Histology analysis

The burn wound tissue was cut into 0.5 × 1 cm size blocks and placed in paraffin embedded cassettes. The tissues were cut into 5 μm paraffin‐embedded sections, which were deparaffinized and rehydrated, followed by HE staining according to the standard procedure. Sections were observed and photographed under a light microscope. Masson staining using Masson Tricolor Staining Kit (G1006, Servicebio) was used to observe the distribution of collagen.

### 2.6 Scanning electron microscopy

Planktonic bacteria were removed from the tissue surface by rinsing three times using sterile saline. After fixation with 2.5% PBS for 24 h at 4°C, samples were rinsed three times with sterile saline and dehydrated using alcohol. After spray‐gold treatment under vacuum, samples were dehydrated and then observed under a scanning electron microscope (Sigma VP40).

### 2.7 qPCR

RNA was isolated from tissue followed by cDNA synthesis for real‐time PCR. Primers were shown in Table 1. PCR conditions were set as follows: 95°C for 10 min, 40 cycles of 95°C for 15 s and 60°C for 1 min. With β‐actin as a control, gene level was analyzed using 2^−ΔΔCt^ method.

**TABLE 1:** Primers used in this study.

Gene	Sequence (5′–3′)	Genetic locus	Length (bp)
β‐Actin	GCAGGAGTACGATGAGTCCG GGGTGTAAAACGCAGCTCAG	NM_031144.3	83
IL‐4	AAGGAACACCACGGAGAACG CAGACCGCTGACACCTCTAC	NM_201270.1	148
IL‐17	CCATGTGCCTGATGCTGTTG GTTATTGGCCTCGGCGTTTG	NM_001106897.1	104
VEGFA	AACGAAAGCGCAAGAAATCCC ATGCTTTCTCCGCTCTGAACA	NM_031836.3	74
TNF‐α	ATGGGCTCCCTCTCATCAGT GCTTGGTGGTTTGCTACGAC	NM_012675.3	106
IFN‐γ	GGCAAAAGGACGGTAACACG TCTGTGGGTTGTTCACCTCG	NM_138880.3	207
IFN‐β1	CTCCAGTTCCGACAAAGCAC TCCTGTAGCTGAGGTTGAGC	NM_019127.1	85

### 2.8 Statistical method

Data were processed by GraphPad Prism 5.0 and displayed as mean ± SD. Comparisons were made using Student's *t*‐test. *p* < 0.05 refers to a difference.

## 3 RESULTS

### 3.1 PRF and PRP promote the healing of burn wounds with biofilm

**FIGURE 1:**
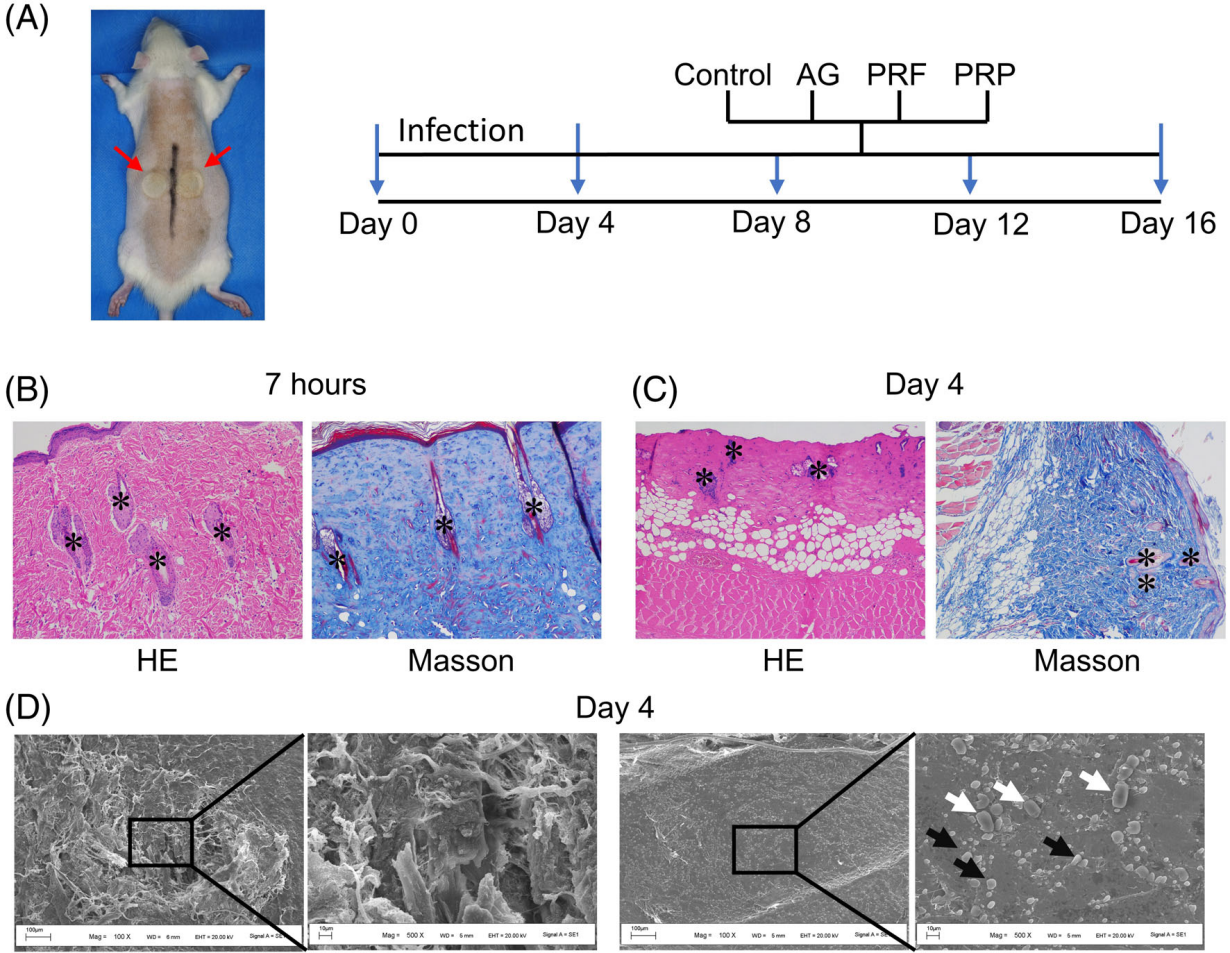
Establishment of an in vivo biofilm model on burn wounds. (A) Time line depicting the burn injury and wound infection model along with control, AG, PRF, and PRP treatments. (B and C) HE and Masson analyses of burn wound on 7 h (B) and day 4 (C) post‐infection of *S. aureus* and *P. aeruginosa*. HE and Masson, ×100. *, hair follicle. (D) SEM images of burn wound on day 4 post‐infection of *S. aureus* and *P. aeruginosa*.

Wound infection was induced by a heated circular copper disk in rats (Figure 1A). Two full‐thickness circular wounds, 2 cm in diameter, were created between the scapulae of the rat. Seven hours after burn injury, the skin structure was normal, and the epidermis was still present (Figure 1B). Collagens were stained blue and keratins were red. *S. aureus* and *P. aeruginosa* were applied to infect the burn wounds. Four days after infection, the burn wounds were infiltrated with biofilm. HE image showed that skin structure was disturbed, and the epidermis was detached (Figure 1C). We also observed that the collagens were loose and disordered compared to 7 h after burn injury (Figure 1B,C), indicating broken and damaged collagen induced by burns. Furthermore, mixed‐species bacteria aggregates were detected by SEM (Figure 1D), suggesting that the dual‐species biofilm model was successfully established.

**FIGURE 2:**
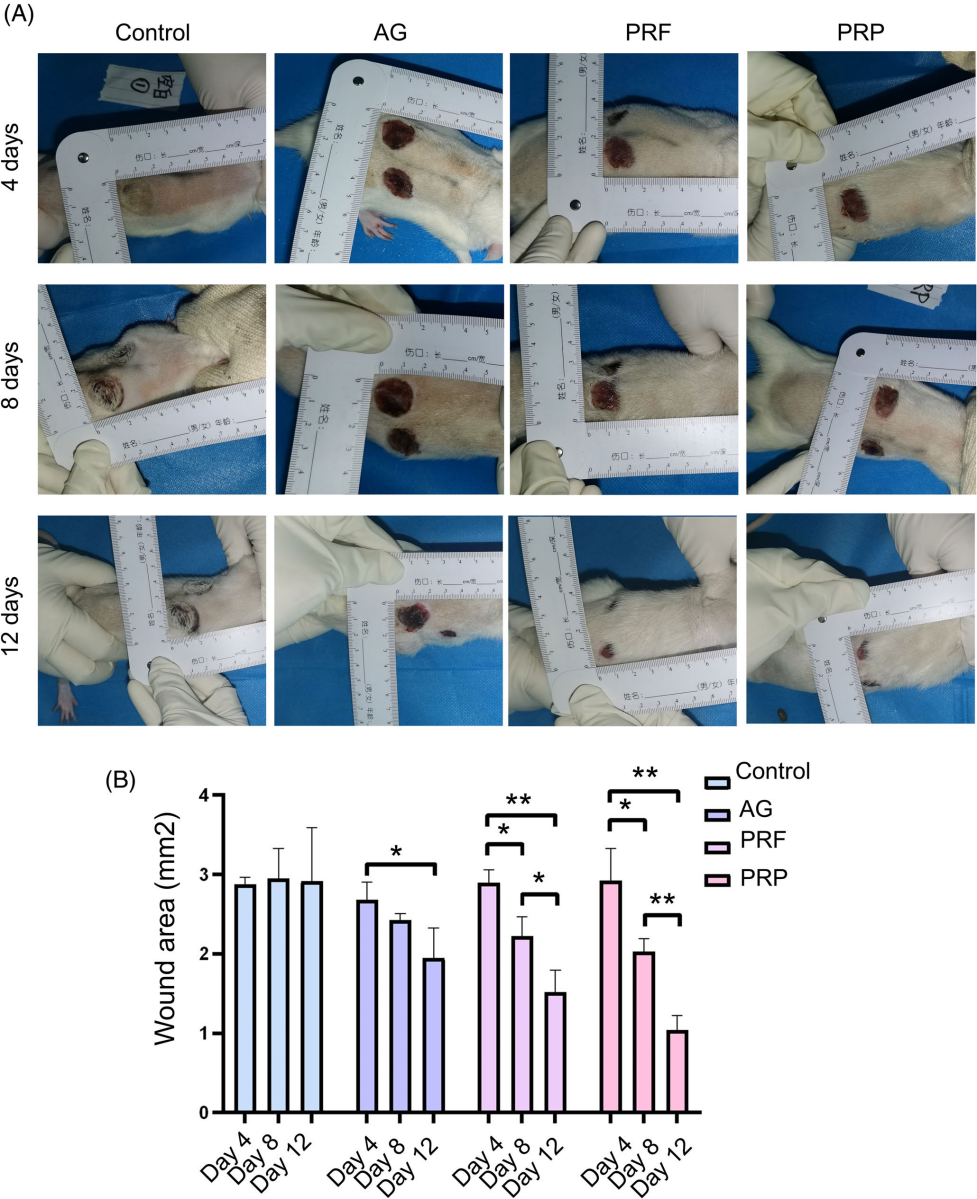
Effect of PRF and PRP on wound healing in biofilm model. (A) AG, PRF, and PRP treatments on burn wounds with biofilm were photographed every 4 days. (B) Quantification of burn wounds with biofilm after treatments. *p < 0.05; **p < 0.001.

**FIGURE 3:**
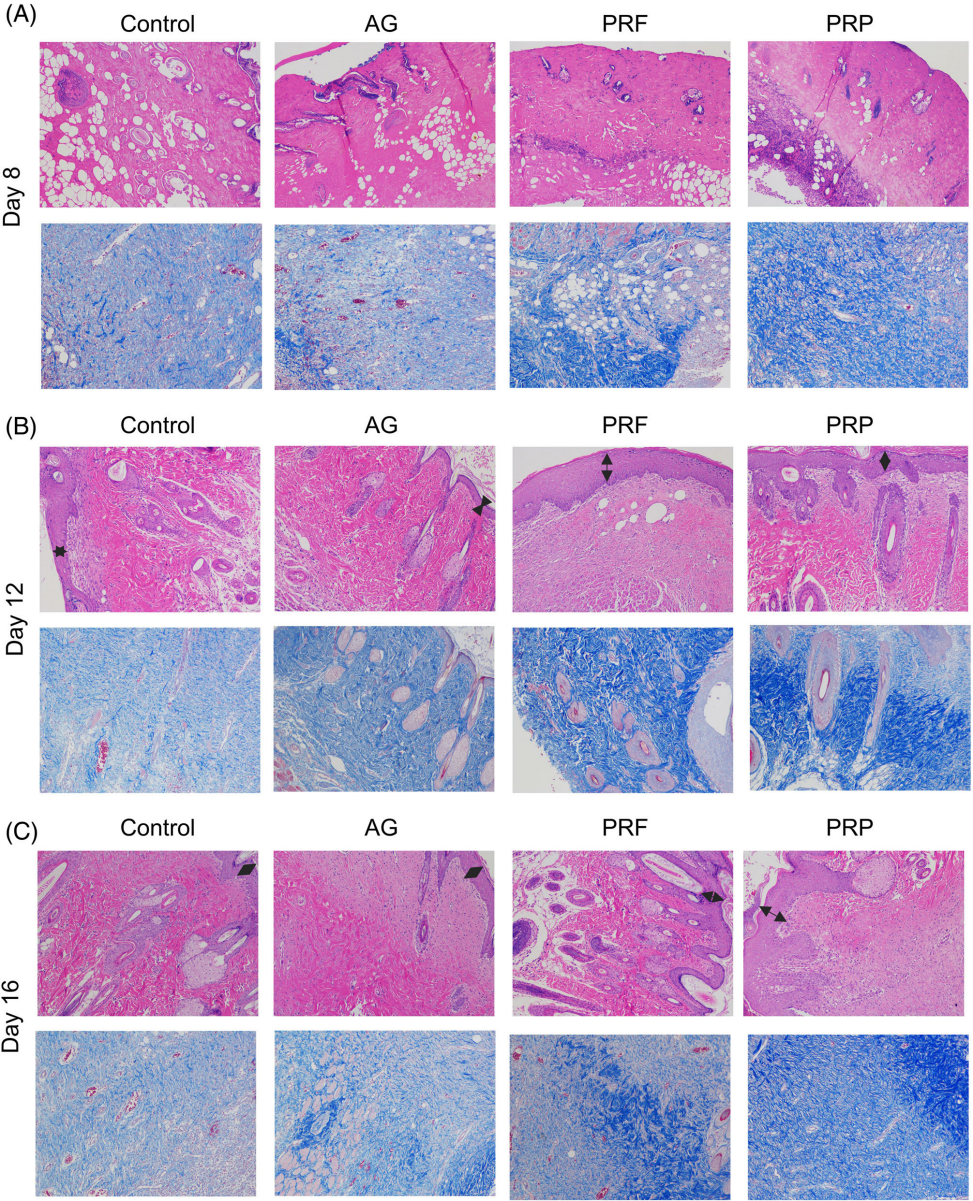
Histology analysis of burn wounds with biofilm. (A–C) HE and Masson analyses showing histological changes of burn wounds with biofilm after treatment at day 8 (A), day 12 (B), day 16 (C) post‐burn injury. HE and Masson, ×100.

To evaluate the effects of PRP and PRF on dual‐species biofilm in burn wounds, rats were assigned into four groups: control, AG, PRF, and PRP groups (Figure 1A). AG, PRF, and PRP dressing were used to cover the burn wounds at day 4 after burn injury. We examined burn wound healing every 4 days, and assessed wound closure in each group. In the control group, the dual‐species biofilm wounds were not healed significantly after 12 days (Figure 2A,B). Treatment with PRF and PRP reduced wound size on day 8 after burn injury, while AG significantly promoted burn wound healing at day 12 (Figure 2A,B). Histological examination showed thickening of the edge of the epidermal layer of PRF‐ and PRP‐treated wounds at days 12 and 16 compared with the control group (Figure 3). Masson images showed that new collagen in the PRF and PRP groups during wound healing began to rearrange at day 8 compared to the control and AG groups (Figure 3). Furthermore, more new collagen fibers were accumulated in the dermis layer in the PRF and PRP groups. These results show that the PRF and PRP groups were able to form new collagen and skin appendages in the shortest possible time, suggesting that they promote skin regeneration and thus accelerate wound healing.

### 3.2 Bacteriostasis effect of PRF and PRP on burn wounds with biofilm

**FIGURE 4:**
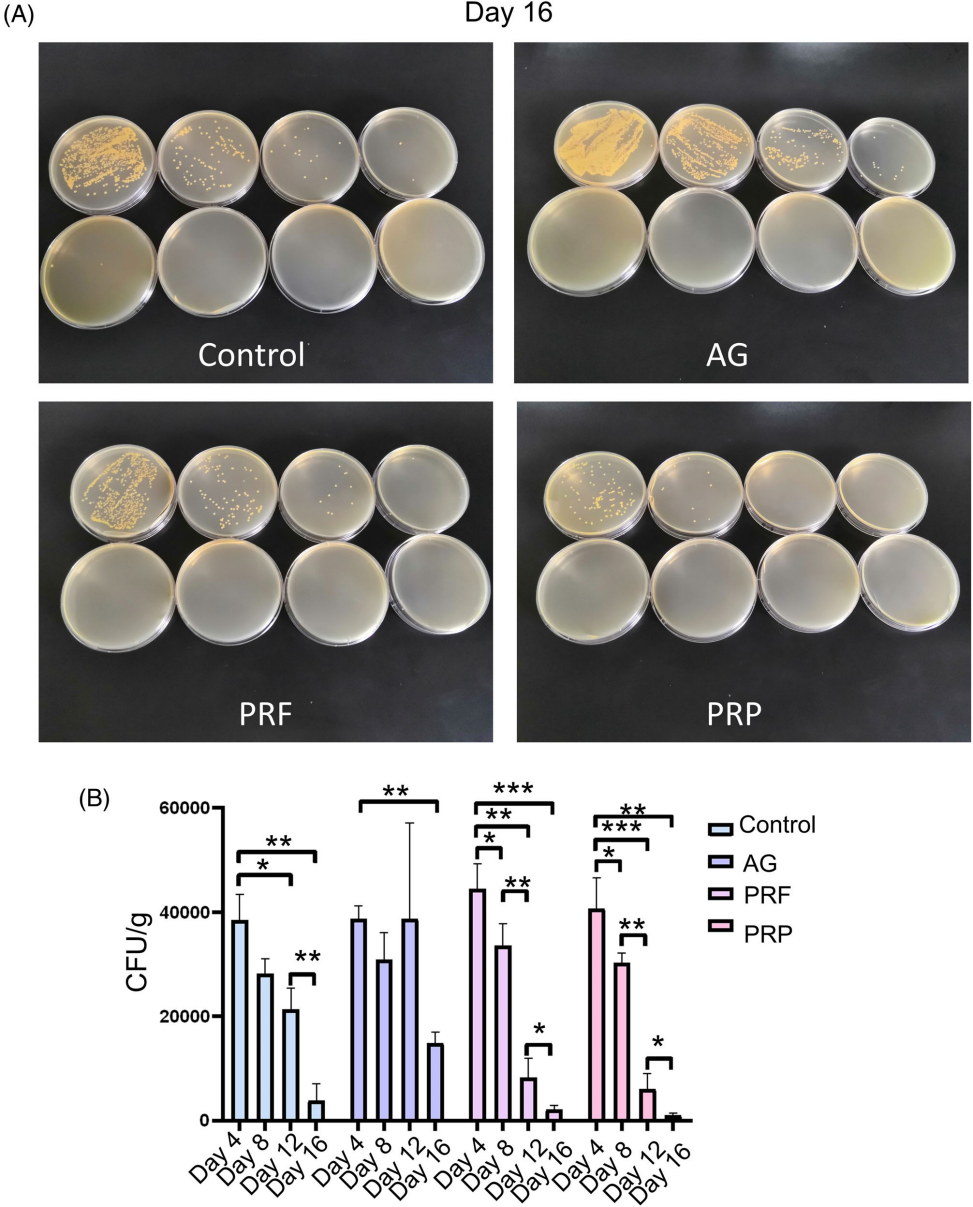
Quantitative skin biopsy bacterial growth in burn wounds with biofilm. (A) Bacterial colonies on the agar plates. (B) Log10 CFU/g counts. *p < 0.05; **p < 0.001, and ***p < 0.0001.

At day 16 after burns and bacterial infections, PRF and PRP had a significant bacteriostatic effect compared to the control group, while AG had a poor bacteriostatic effect (Figure 4A,B). *S. aureus* and *P. aeruginosa* counts decreased after 4 days of PRF and PRP treatment (Figure 4B), but the number of mixed bacteria in the AG‐treated wound area did not decrease. Bacterial counts remained high in the AG treatment group after 8 days of treatment. These results indicate that PRF and PRP accelerated the reduction of bacterial counts in wounds with *S. aureus* and *P. aeruginosa* biofilm, whereas AG did not have a favorable bacterial inhibitory effect on the dual‐species biofilm in burn wounds.

**FIGURE 5:**
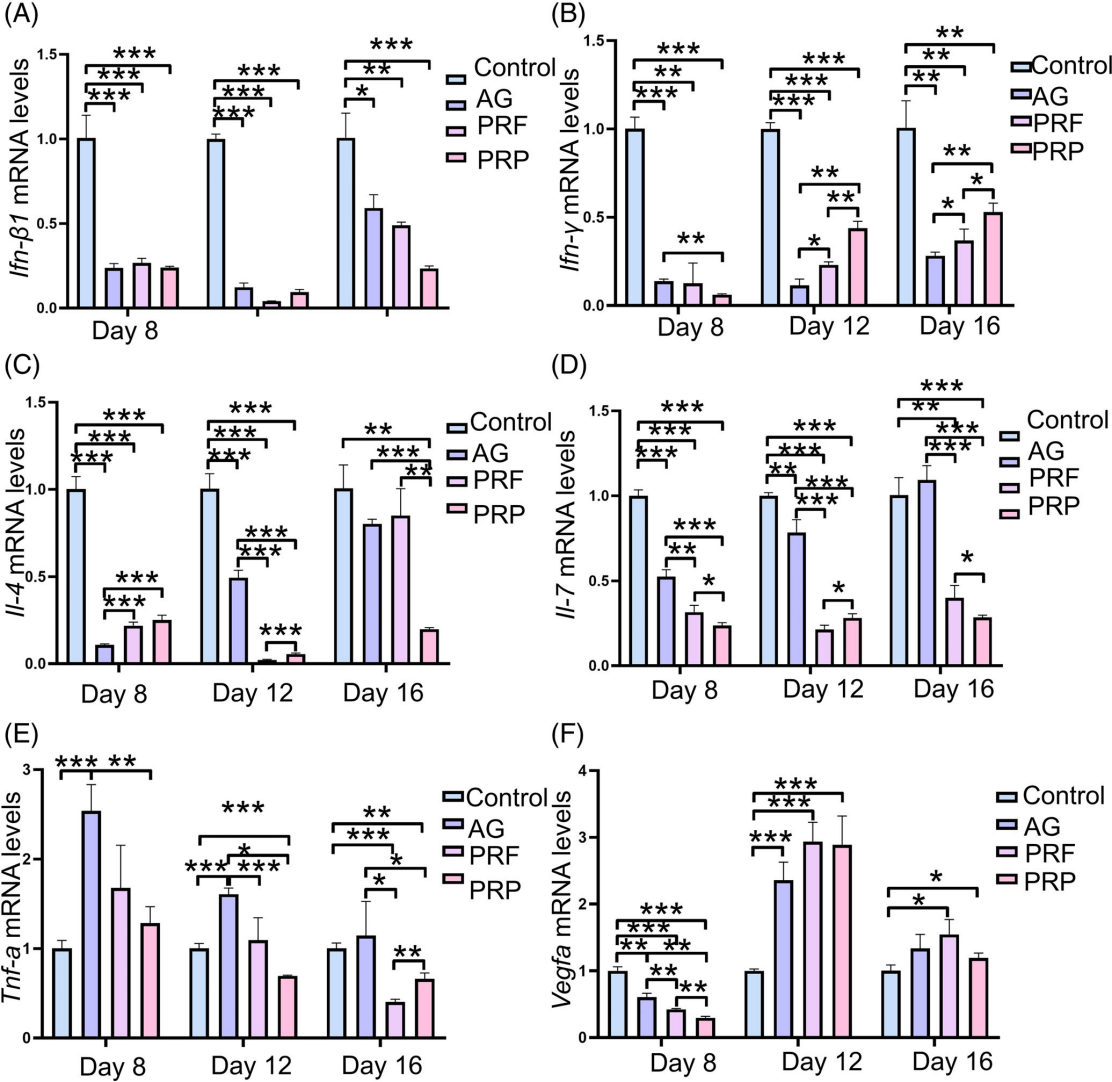
Dermal expression of burn‐induced inflammatory mediators and VEGFA. mRNA expression of inflammatory mediators and VEGFA in burn wounds with biofilm after treatments. *p < 0.05; **p < 0.001, and ***p < 0.0001.

PRF and PRP reduce inflammatory mediators and favor angiogenesis in burn wounds with biofilms. To investigate whether PRF and PRP modulate the wound environment through the release and activity of various cytokines, the levels of cytokines in burn wounds were quantified using qPCR. AG, PRF, and PRP significantly reduced the mRNA level of interferon (IFN)‐β1 and IFN‐γ compared to control (Figure 5A,B). PRP significantly suppressed mRNA level of IL‐4 on days 8, 12, and 16 post‐burn injury, while PRF and AG significantly restrained mRNA level of IL‐4 at day 8 and day 12 (Figure 5C). Treatment with PRP, PRF, and AG significantly reduced mRNA level of IL‐7 at day 8 and day 12, while at day 16 post‐burn injury, only PRF and AG treatments decreased mRNA level of IL‐7 in burn wounds (Figure 5D). TNF‐α was significantly increased in AG‐treated wounds at day 8 and day 12 post‐burn injury, while it was significantly decreased in PRF‐treated wounds at day 16 and in PRP‐treated wounds at day 12 and day 16 post‐burn injury (Figure 5E). Interestingly, VEGFA mRNA expression was significantly down‐regulated in AG‐, PRF‐, and PRP‐treated wounds compared to control at day 8 post‐burn injury, while it was significantly up‐regulated in AG‐, PRF‐, and PRP‐treated wounds at day 12 (Figure 5F). At day 16, mRNA level of VEGFA in PRF‐ and PRP‐treated wounds was still significantly higher than control (Figure 5F). These results suggest that AG, PRF, and PRP treatments could reduce inflammatory mediators and induce VEGFA, while AG treatment increases pro‐inflammatory cytokine TNF‐α in dual‐species biofilm burn wounds.

## 4 DISCUSSION

Biofilms in wounds are formed when certain microorganisms attach to the wound surface.^13^ Biofilms are resistant to antimicrobial agents and key host defenses, highlighting the importance of treating biofilm infections in burn wounds.^14^, ^15^, ^16^ Biofilms are a major problem in burns, accounting for 60% of burn‐related deaths.^14^ Biofilms also inhibit epithelialization and granulation tissue formation and enhance the chronic inflammatory response to wounds.^17^ Bacteria in biofilms are more resistant to antimicrobial agents than planktonic cells because the biofilm environment provides several different defense mechanisms.^18^ Therefore, the effective strategies for treatment of bacterial biofilms in burn wounds are urgently required.

Burn wounds become rapidly colonized by *S. aureus* and colonize within hours to days.^14^ This study used *S. aureus* and *P. aeruginosa* to infect burn wounds in rats to establish a dual‐species biofilm infection model. After 4 days of infection, aggregation of mixed‐species bacteria was observed by SEM. AG, PRF, and PRP have been reported to promote wound healing and can be used to treat chronic wounds.^8^, ^19^, ^20^, ^21^ However, a comprehensive analysis of AG, PRF, and PRP on dual‐species biofilm in burn wound is lacking. Here, we demonstrated the roles of AG, PRF, and PRP in the closure, re‐epithelialization, bacterial growth, inflammation, and angiogenesis of dual biofilm burn wounds, which coordinately contribute to wound healing.

It was observed that PRF and PRP significantly accelerated wound closure in a short period of time compared to AG. PRF and PRP significantly increased the thickness and length of neoepidermal tissue, thereby re‐epithelializing the burn wound. AG significantly promoted wound closure until day 12 after burn and infection. Bacteria in biofilms may delay wound healing by impeding epithelialization and inducing an inflammatory response in chronic wounds.^17^ Notably, PRF and PRP reduced the number of bacteria in the wound faster than the control and AG‐treated groups at all three time points. These results suggest that PRF and PRP have a strong antimicrobial effect on the dual‐species biofilm of burn wounds, whereas AG does not. PRP and PRF therapy is an endogenous therapeutic technique. The mechanisms of antimicrobial action of PRP and PRF depend on its components. Seven thrombin‐releasable antimicrobial peptides from human platelets are identified.^22^ PRF might be applied to treat postoperative infections caused by biofilm producing oral staphylococcus.^11^ In this study, PRF and PRP showed bactericidal activity against dual biofilms in burn wounds, suggesting that they could be potential antimicrobial therapies for burn wounds with biofilms.

The inflammatory response following tissue injury is the first stage in the wound healing process.^23^ IL‐4 and IL‐13 belong to the class II inflammatory response.^24^ Acute inflammatory response is triggered by Th2 activity, characterized by IL‐4, IL‐5, IL‐13, and IFNγ.^25^ These Th2 cytokines can facilitate *S. aureus* colonization since they impair skin barrier.^26^, ^27^ Furthermore, IL‐4 inhibits intracellular killing of *S. aureus* in infected macrophages in vitro.^28^ IFN‐γ supports the growth of *S. aureus* strains in vitro.^25^ Interestingly, we observed that AG, PRF, and PRP reduced the mRNA levels of IL‐4 and IL‐13 as well as IFN‐γ in wound tissues. We observed reduced TNF‐α expression in PRF‐ and PRP‐treated groups at day 16 after burn injury and infection compared to control group, while it was significantly increased in AG‐treated group. Considering that the bacterial counts in the AG‐treated group were significantly higher than those in the control group, we concluded that AG had poor bactericidal activity against *P. aeruginosa*. Our data show that PRF and PRP treatments significantly reduced inflammatory mediators that promote the growth of *S. aureus* and *P aeruginosa*‐stimulated TNF‐α in wound tissue.^29^

VEGFA is a key mediator of physiological and pathological angiogenesis.^30^ It has been reported that injection of PRP increases VEGF level during the healing process from deep dermal burns.^31^ Here, we found that mRNA level of VEGFA of wound tissues of AG‐, PRF‐, and PRP‐treated groups was significantly increased at day 12 post‐burn injury and infection. At day 16, mRNA level of VEGFA in PRF‐ and PRP‐treated groups was significantly higher than control group. These data indicate that PRF and PRP effectively stimulate the secretion of VEGF, promoting angiogenesis in wounds with biofilm.

The study's findings are constrained by its experimental design and scope. The dual‐species biofilm model, while insightful, does not encompass the polymicrobial nature of many human burn wound infections, which may involve multiple bacterial and fungal species. This could limit the applicability of the results to clinical settings where infections are more diverse. Moreover, the study did not account for the potential impact of systemic factors such as the patient's overall health, comorbidities, and immune status, which can significantly influence wound healing and response to treatment. Additionally, the concentration and application frequency of PRF and PRP were controlled and consistent; in clinical practice, these factors can vary, potentially affecting the treatment effectiveness. Finally, this study was conducted in a controlled laboratory setting and does not fully reflect the environmental challenges faced by burn wounds in a clinical setting, such as exposure to other microbial contaminants and different methods of care. These factors must be considered when applying the results of these studies to human patients.

## 5 CONCLUSION

In this study, a dual‐species biofilm model community for *S. aureus* and *P. aeruginosa* was established after burn injury in rats. PRF and PRP accelerated the healing of dual‐species biofilm‐infected wounds with good antimicrobial efficacy against *S. aureus* and *P. aeruginosa*.

## CONFLICT OF INTEREST STATEMENT

The authors declare no conflict of interest.

## ETHICS STATEMENT

All animal experiments were complied with the ARRIVE guidelines and performed in accordance with the National Institutes of Health Guide for the Care and Use of Laboratory Animals. The experiments were approved by the Institutional Animal Care and Use Committee of General Hospital of Southern Theater Command of PLA (No. 2022‐GZ524).

## Data Availability

The data that support the findings of this study are available from the corresponding author upon reasonable request.
